# Public health round-up

**DOI:** 10.2471/BLT.19.010519

**Published:** 2019-05-01

**Cyclone Idai**

A health worker vaccinates children against cholera as part of the World Health Organization’s response to Cyclone Idai.

**Figure:**
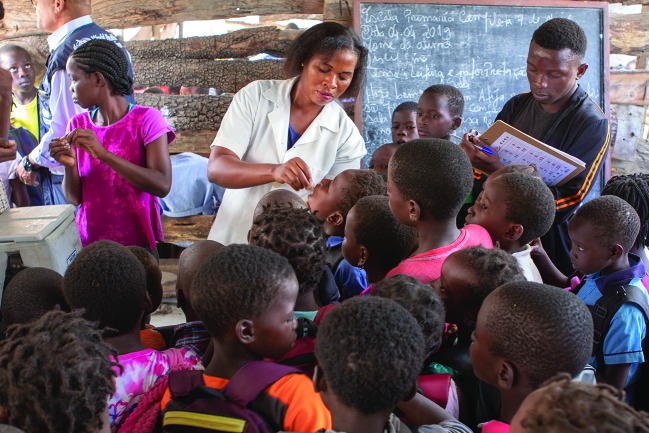
WHO/Mark Nieuwenhof

## Cyclone Idai response

The Ministry of Health in Mozambique concluded a successful six-day emergency cholera vaccination campaign as part of a response to the health emergency caused by Cyclone Idai on 10 April. The cyclone made landfall near the city of Beira on 15 March.

The vaccines were donated by Gavi, the Vaccine Alliance, from the global cholera vaccine stockpile and arrived in Beira on 2 April. Distribution was assured by approximately 1200 community volunteers who reached more than 800 000 people in the four districts affected by the cyclone.

The campaign was supported by partners including the World Health Organization (WHO), the United Nations Children's Fund, (UNICEF), Médecins Sans Frontières (MSF), International Federation of the Red Cross and Red Crescent Societies (IFRC) and Save the Children.

In Mozambique, WHO has also deployed experts, including epidemiologists, logisticians and disease-prevention experts, and has helped with the distribution of supplies, including intravenous fluids, diagnostic tests, oral rehydration solution and other medical supplies to support cholera treatment centres.

WHO is also supporting health response efforts in Malawi and Zimbabwe, which were also affected by heavy rains and flooding. According to United Nations Office for the Coordination of Humanitarian Affairs some 1.6 million people in the three countries have been affected by the cyclone.

In Malawi, WHO has sent medicines and medical supplies to meet the needs of tens of thousands of people and is continuing to support six district outreach clinics. In Zimbabwe, medical supplies are being delivered to health facilities in the affected areas and eleven satellite clinics have been established to provide emergency health services to people who cannot access the pre-existing health facilities. 

https://www.who.int/emergencies/cyclone-idai/events-as-they-happen

## WHO convenes experts on human genome editing

WHO’s new expert advisory committee on developing global standards for governance and oversight of human genome editing met for the first time on 18-19 March. The committee agreed that given the current state of knowledge, it would be irresponsible at this time for anyone to proceed with clinical applications of human germline genome editing and called for WHO to establish a central registry on human genome editing research.

The committee invited input from scientists conducting human genome editing research on the technical environment and current governance arrangements that relate to work in this area. Over the next two years, the committee will consult a wide range of stakeholders and provide recommendations for a comprehensive genome editing governance framework that is scalable, sustainable and appropriate for use at the international, regional, national and local levels. The committee will solicit the views of multiple stakeholders including patient groups, civil society, ethicists and social scientists.

https://www.who.int/news-room/detail/19-03-2019-who-expert-panel-paves-way-for-strong-international-governance-on-human-genome-editing

## Ebola cases exceed 1000

A total of 1186 confirmed and probable Ebola virus disease cases had been reported in the Democratic Republic of the Congo as of 9 April. Some 38 of 130 affected health areas were reporting active transmission. The risk of national and regional spread remains very high.

Response efforts have included the vaccination of 96 000 people in the Democratic Republic of the Congo, along with health workers in Uganda and South Sudan. Over 44 million border screenings have been done. Thus far, no cases have spread beyond North Kivu province which borders Uganda and Rwanda, and Ituri province, which borders Uganda and South Sudan.

WHO Director-General Tedros Adhanom Ghebreyesus called for donor support to meet the financial requirements of the response on 23 March. “Despite the increased frequency of attacks by armed groups, WHO will stay the course and will work with communities to end this outbreak together with the Ministry of Health and partners,” he said. “We need redoubled support from the international community, and a commitment to push together to bring this outbreak to an end.”

https://www.who.int/news-room/detail/23-03-2019-who-reaffirms-commitment-to-the-democratic-republic-of-the-congo-as-ebola-outbreak-nears-1-000-cases-amid-increased-violence

## Islamic Republic of Iran flood response

WHO airlifted essential medical supplies to the Islamic Republic of Iran on 10 April to respond to the health needs of thousands of people displaced by heavy flooding caused by extreme rainfall, which began on 19 March. 

Twenty-eight of the country’s 31 provinces have been affected by the floods, causing widespread damage to municipal facilities, including health centres and hospitals. Many health facilities in affected areas are either fully or partially damaged, and the risk of water- and vector-borne diseases, including malaria, cholera and other communicable diseases is increasing, also due to the interruption of the water supply.

http://www.emro.who.int/irn/iran-news/who-airlifts-medical-supplies-for-flood-response-in-islamic-republic-of-iran.html

## Afghanistan polio campaign

Afghanistan began the first round of a national polio immunization campaign on 1 April.

The 5-day campaign will target over 9.3 million children under-5 years of age in nearly all of Afghanistan’s provinces.

During the campaign, about 8.4 million children aged between 6 months to 5 years will also be given vitamin A capsules.

http://www.emro.who.int/afg/afghanistan-news/around-93-million-children-to-be-vaccinated-in-first-round-of-nids-in-2019.html

## Resistant TB in Europe

Just over one in four (28%) people with new or relapsed tuberculosis (TB) infections in eastern Europe were multi-drug resistant in 2017, according to the latest report by WHO and the European Centre for Disease Prevention and Control (CDC).

The report, entitled *Tuberculosis surveillance and monitoring in Europe 2019*, was published 22 March.

It found that of the 275 000 people with new or relapsed tuberculosis in the WHO European Region in 2017, an estimated 77 000 patients have multidrug-resistant tuberculosis (resistant to rifamipicin and isoniazid), while almost 7000 patients have extensively drug-resistant tuberculosis (XDR–TB; resistant to all first- and most second-line drugs).

http://www.euro.who.int/en/health-topics/communicable-diseases/tuberculosis/news/news/2019/3/drug-resistant-strains-could-become-the-dominant-form-of-tb-in-europe-its-time-to-end-tb

**Cover photo**

An aerial view shows damage from the flood waters after Cyclone Idai, in Bangula town, Malawi. Cyclone Idai has caused floods in Malawi, Mozambique and Zimbabwe, leaving more than 1000 people dead and thousands more missing.

**Figure:**
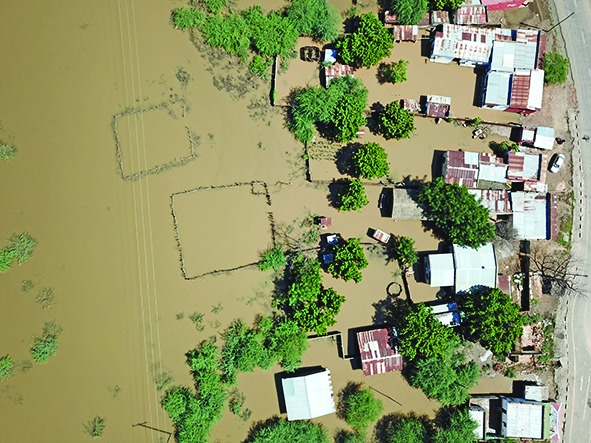
UNICEF/Juskauskas, Kunje & Chipukunya

## Health worker killings

WHO condemned the killing of health workers in Libya and Pakistan on 8 and 9 April.

In Libya, two doctors were killed while providing critical services to civilians in Tripoli. Dr Ahmed Al Mandhari, WHO Regional Director for the Eastern Mediterranean said,“These doctors risked their lives to evacuate wounded patients from conflict areas and targeting them and health facilities at such times, worsens the situation for civilians caught up in conflict.” WHO has documented more than 46 attacks on health workers and facilities throughout 2018–2019 across Libya.

In Pakistan,****Mr Wajid Ali was shot and killed on 8 April while supporting polio eradication and immunization efforts in Khyber Pakhtunkhwa. Dr Al-Mandhari stated that WHO and its partners will nevertheless continue efforts to work with the Government of Pakistan, UNICEF, and other partners to eradicate polio and ensure the highest possible level of health for all the people of Pakistan. 

http://www.emro.who.int/pak/pakistan-news/who-condemns-killing-of-polio-worker-in-pakistan.html

http://www.emro.who.int/lby/libya-news/who-condemns-the-killing-of-doctors-in-the-libyan-conflict.html

## Life expectancy rises

Between 2000 and 2016, global life-expectancy at birth increased from 66.5 to 72.0 years according to WHO’s *World health statistics 2019: monitoring health for the SDGs* which was published on 4 April. Healthy life expectancy at birth – the number of years one can expect to live in full health – was reported to have increased from 58.5 years in 2000 to 63.3 years in 2016.

Life expectancy remains strongly affected by income. In low-income countries, life expectancy is 18.1 years lower than in high-income countries. The report estimates that one child in every 14 born in a low-income country will die before their fifth birthday.

https://apps.who.int/iris/bitstream/handle/10665/311696/WHO-DAD-2019.1-eng.pdf

## WHO and partners call for collaboration on global health

WHO, governments and global health leaders have called for improved partnerships and resourcing to support WHO’s mission to deliver care, services and protection for billions of people by 2023. An inaugural two-day WHO Partners Forum, took place in Stockholm on 9-10 April, bringing together global leaders in health and development, representing the public sector, health partnerships and non-state actors. The forum was an opportunity to discuss ways to meet WHO’s resource needs under the Organization’s 13^th^ General Programme of Work. The meeting resulted in a shared understanding of how to strengthen partnerships and improve the financing of WHO, with an emphasis on predictability and flexibility.

https://www.who.int/news-room/detail/09-04-2019-inaugural-who-partners-forum-launches-new-push-for-collaboration-on-global-health

**Looking ahead**

22 – 26 June - International Epilepsy Congress. Bangkok, Thailand

1 – 5 July - World Conference of Science Journalists. Lausanne, Switzerland.

4 – 7 July - Sexually Transmitted Infections Conference. Vancouver, Canada.

26 – 28 August - 68^th^ UN Civil Society Conference. Salt Lake City, USA.

